# Disruption of the Suprachiasmatic Nucleus Blunts a Time of Day-Dependent Variation in Systemic Anaphylactic Reaction in Mice

**DOI:** 10.1155/2014/474217

**Published:** 2014-04-17

**Authors:** Yuki Nakamura, Kayoko Ishimaru, Yu Tahara, Shigenobu Shibata, Atsuhito Nakao

**Affiliations:** ^1^Department of Immunology, Faculty of Medicine, University of Yamanashi, 1110 Shimokato, Chuo, Yamanashi 409-3898, Japan; ^2^Department of Physiology and Pharmacology, School of Advanced Science and Engineering, Waseda University, 2-2 Wakamatsu-cho, Shinjuku-ku, Tokyo 162-8480, Japan; ^3^Atopy Research Center, Juntendo University School of Medicine, 2-1-1 Hongo, Bunkyo-ku, Tokyo 113-8421, Japan

## Abstract

Anaphylaxis is a severe systemic allergic reaction which is rapid in onset and potentially fatal, caused by excessive release of mediators including histamine and cytokines/chemokines from mast cells and basophils upon allergen/IgE stimulation. Increased prevalence of anaphylaxis in industrialized countries requires urgent needs for better understanding of anaphylaxis. However, the pathophysiology of the disease is not fully understood. Here we report that the circadian clock may be an important regulator of anaphylaxis. In mammals, the central clock located in the suprachiasmatic nucleus (SCN) of the hypothalamus synchronizes and entrains peripheral circadian clock present in virtually all cell types via neural and endocrine pathways, thereby driving the daily rhythms in behavior and physiology. We found that mechanical disruption of the SCN resulted in the absence of a time of day-dependent variation in passive systemic anaphylactic (PSA) reaction in mice, associated with loss of daily variations in serum histamine, MCP-1 (CCL2), and IL-6 levels. These results suggest that the central SCN clock controls the time of day-dependent variation in IgE-mediated systemic anaphylactic reaction, which may provide a novel insight into the pathophysiology of anaphylaxis.

## 1. Introduction

Anaphylaxis is a rapid, potentially fatal, multiorgan system, allergic reaction caused by the excessive release of mediators including histamine and cytokines/chemokines from mast cells and basophils upon allergen/IgE stimulation [1]. It typically causes a number of symptoms including an itchy rash, throat swelling, and low blood pressure associated with low body temperature. A recent nationwide cross-sectional telephone survey in USA estimated that the prevalence of anaphylaxis in the general adult population is at least 1.6% and medications were the most common trigger (35%), followed by foods (32%) and insect stings (19%) [2]. It is also recently reported that an estimated 1 in 300 of the European population at some time in their lives is affected [3]. Given the increased prevalence of anaphylaxis in industrialized countries, more basic research is urgently needed for better understanding of anaphylaxis that leads to efficient prevention of the disease. However, the pathophysiology of anaphylaxis is not fully understood.

In mammals, the internal time keeping system “circadian clock” drives the daily rhythms in behavior and physiology (e.g., sleep-wake cycles, body temperature, blood pressure, and hormonal secretions) that enable the organisms to keep track of the time of day according to daily changes in light intensity [4, 5]. Mammalian circadian clock system consists of the central oscillator located in the suprachiasmatic nucleus (SCN) of the hypothalamus and peripheral oscillators present in virtually all cell types [4, 5]. Light activates a specific group of photoreceptors in the retina connected to the central SCN clock which synchronizes and entrains peripheral circadian clock via neural and endocrine pathways. The molecular mechanisms of rhythm generation are cell autonomous, highly conserved in the SCN and peripheral cells, and created and maintained by interlocked transcriptional-translational feedback loops consisting of several “clock genes” and their protein products [4, 5].

We have recently shown that a passive systemic anaphylactic (PSA) reaction in mice exhibits a time of day-dependent variation, relying on the normal activity of a key clock gene,* Period2* (*Per2*) [6], suggesting that the canonical clock gene is required for the daily rhythm generation observed in PSA reaction. However, it remains obscure whether the daily rhythms are generated precisely by the circadian clock system or not, since* Per2* has nonclock functions and may regulate the daily rhythms independently of the circadian clock system [4, 5].

Therefore, this short study aimed to clarify the precise role of the circadian clock system in the generation of daily rhythms in anaphylaxis. For this purpose, we examined the effects of SCN ablation on the time of day-dependent variations in PSA reaction since SCN ablation reliably eliminates the normal activity of the circadian clock system [4, 5].

## 2. Materials and Methods

### 2.1. Mice

Male 8–12-week-old ICR mice (Japan SLC, Tokyo, Japan) were bred under specific pathogen-free conditions and 12-hour light/12-hour dark conditions (L/D cycles; the light was turned on at 6:00 a.m., Zeitgeber time (ZT) 0, and the light was turned off at 6:00 p.m., ZT12) with* ad libitum* access to food and water, for at least 2 weeks. All animal experiments were approved by the Institutional Review Board of University of Yamanashi and Waseda University.

### 2.2. SCN Disruption

Bilateral thermal lesions of the SCN were obtained stereotactically (Narishige Co., Tokyo, Japan) under anaesthesia as described previously [7]. One month after the surgery, we selected animals with complete lesioning of the SCN after confirmation of arrhythmic general locomotor activity recorded with an infrared radiation sensor (F5B, Omron, Tokyo, Japan) and analyzed with CLOCKLAB software (Actimetrics, Wilmette, IL, USA) as described previously [7]. A histological check of lesion sites was also performed after finishing the PSA experiments using Nissl staining.

### 2.3. Passive Systemic Anaphylactic (PSA) Reaction

A passive systemic anaphylactic (PSA) reaction was induced in mice as previously described with some modifications [8]. Briefly, mice were sensitized with an intravenous injection of 20 *μ*g with mouse anti-TNP IgE in 0.2 mL PBS. The mice were intravenously challenged with 1000 *μ*g DNP-BSA in 0.2 mL PBS 24 hours later. Their rectal temperature was measured with a digital thermometer (Shibaura Electronics, Tokyo, Japan) every 5 minutes from 20 minutes before DNP-BSA challenge and thereafter until 120 minutes after the start of the examination. The rectal temperature was also measured at 180 minutes after the start of the examination.

### 2.4. Serum MCP-1 (CCL2), IL-6, and Histamine Levels

Serum samples were collected from the mice at 10 minutes or 180 minutes after induction of PSA reactions. The amounts of MCP-1 (CCL2) and IL-6 in the serum at 180 minutes after induction of PSA reaction were measured using the mouse MCP-1 and IL-6 ELISA kits (R&D, Minneapolis, MN). The amounts of histamine in the serum at 10 minutes and 180 minutes after induction of PSA were measured using histamine EIA kit (Oxford Biomedical research, Inc., Oxford, MI, USA).

### 2.5. Serum IgE and Corticosterone Levels

Serum levels of total IgE and corticosterone were determined by using the mouse IgE ELISA kit (R&D, Minneapolis, MN, USA, or Morinaga Institute of Biological Science, Kanagawa, Japan) or AssayMax Corticosterone ELISA kit (AssayPro, Charles, MO, USA), respectively.

### 2.6. Statistics

Data were expressed as the mean ± S.D. Statistical analysis was performed using an unpaired Student's *t*-test to compare data in different groups. *P* value < 0.05 was considered to indicate a statistically significant difference.

## 3. Results and Discussion

**Figure 1:**
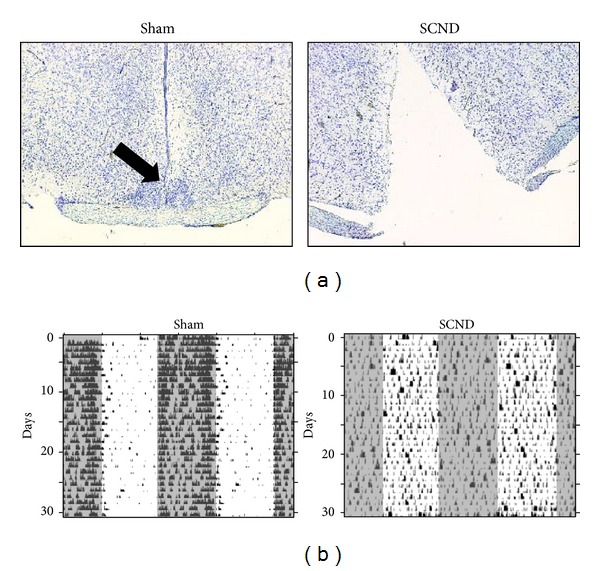
Histological and general locomotor analysis after induction of the bilateral electrolytic lesions of the SCN in mice. (a) The SCN of the mice was destroyed by bilateral electrolytic lesions and complete SCN destruction was ascertained by a postmortem histological analysis. Representative pictures of the staining in sham-operated mice (Sham) and mice with disrupted SCN (SCND) are shown. The arrow indicates the SCN area. (b) General locomotor activity of mice housed under LD 12 : 12 conditions was recorded and representative double-blot actograms of the drinking behavior of the mice during 30 days are shown. The black dots indicate the mouse locomotor activity. Horizontal open and solid bars indicate day and night, respectively. Please note that the drinking behavior (usually accompanied by food intake) became arrhythmic in mice with disrupted SCN (SCND).

In order to clarify the precise role of the circadian clock system in the generation of daily rhythms in systemic anaphylactic reaction, we examined the effects of SCN ablation on a time of day-dependent variations in passive systemic anaphylactic (PSA) reaction in mice, which is a representative model of anaphylaxis. We confirmed complete disruption of the SCN in mice by a histological check of lesion sites by Nissl staining after finishing all the experiments (Figure 1(a)) and by examining behavioral patterns (general locomotor activity with arrhythmicity) (Figure 1(b)).

**Figure 2:**
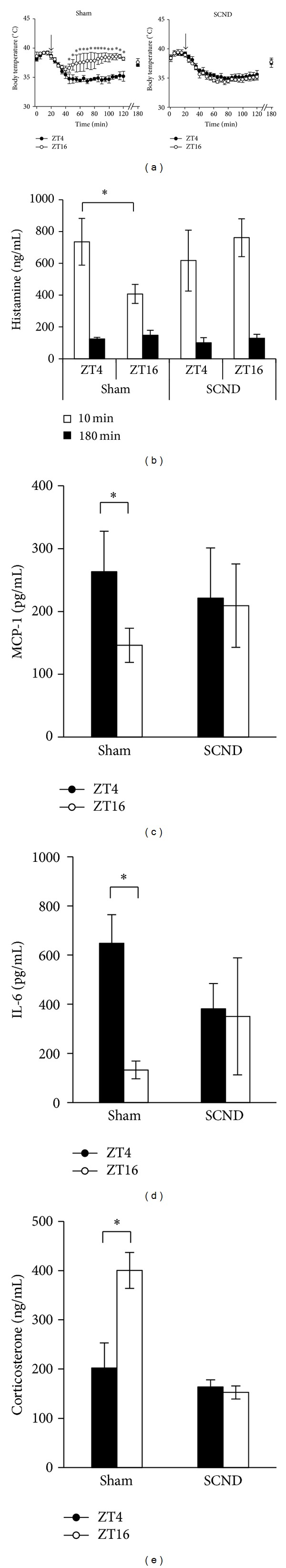
The SCN ablation blunts a time of day-dependent variation in PSA reaction. (a) Changes in rectal temperature over time after the allergen challenge at ZT4 (10:00 a.m.) and ZT16 (10:00 p.m.) in sham-operated mice (Sham) and mice with disrupted SCN (SCND) (*n* = 4). The arrows indicate the time of antigen challenge (b)–(d). The serum histamine (b), MCP-1 (CCL2) (c), and IL-6 (d) levels following induction of PSA reactions at ZT4 and ZT16 in sham-operated mice or mice with SCN disruption (*n* = 4 per group). Concentrations of histamine were measured using serum samples collected at 10 and 180 minutes after induction of PSA reaction at ZT4 or ZT10. Concentrations of MCP-1 and IL-6 were measured using serum samples collected at 180 minutes after induction of PSA reaction at ZT4 or ZT16. (e) The serum corticosterone levels at ZT4 and ZT16 in sham-operated mice and mice with SCN disruption (*n* = 4 per group). *P < 0.05. Similar results were obtained in at least two independent experiments.

The kinetics of PSA reaction was then compared in sham-operated mice and mice with disrupted SCN (Figure 2(a)). Sham-operated mice challenged with the antigen at 10:00 pm (ZT16) showed a smaller drop in the extent of rectal temperatures than the mice challenged at 10:00 am (ZT4). However, such a time of day-dependent variation in PSA reaction was absent in mice with disrupted SCN. Consistently, serum histamine, MCP-1 (CCL2), and IL-6 levels following the induction of the PSA reactions showed similar time of day-dependent variations to the PSA reactions in sham-operated mice, but the variations were absent in mice with disrupted SCN (Figures 2(b)–2(d)). The daily variations in serum corticosterone levels observed in sham-operated mice were also absent in mice with disrupted SCN (Figure 2(e)). In contrast, serum total IgE levels were comparable between sham-operated mice and mice with disrupted SCN (data not shown). These results indicated that disruption of the central clock SCN blunted a time of a day-dependent variation in PSA reaction in association with a loss of rhythmic secretion of corticosterone. These findings suggest that the circadian clock system may contribute to the generation of daily rhythms in anaphylaxis.

Body temperature is under circadian control [4, 5]. However, it appears that the basal body temperatures at ZT4 and ZT16 before induction of PSA (0*∼*20 minutes before the antigen challenge) were comparable in control sham-operated mice (Figure 2(a)). We speculate that this discrepancy might be caused, at least in part, by the experimental manipulation for measuring body temperature. Direct insertion of a digital thermometer into the rectum every 5 minutes likely affects mouse activity in the resting phase and might upregulate the body temperature. In contrast, a time of day-dependent variation in PSA reaction was clearly observed at ZT4 and ZT16 in control sham-operated mice (Figure 2(a)). Thus, it appears that the extent of a time of day-dependent systemic mast cell responses overrides the possible effects of the experimental manipulation on body temperature.

The surgery for SCN ablation may destroy other important nuclei located in this brain area such as temperature center and corticosterone releasing hormone (CRH) neurons. We observed that body temperatures at 180 minutes after the examination were comparable between sham-operated mice and mice with disrupted SCN (Figure 2(a)), suggesting that the brain area for temperature regulation was functional in mice with disrupted SCN. We also observed that basal levels of serum corticosterone were comparable between sham-operated mice and mice with disrupted SCN (Figure 2(a)), suggesting that the brain area for CRH regulation was functional in mice with disrupted SCN (Figure 2(e)). These findings may support the specificity of the surgery to the SCN although we cannot completely exclude some of the nonspecific influences of the SCN ablation on the current results.

It remains to be determined precisely how the dysfunction of the circadian clock (disruption of the SCN) affects the time of day-dependent variation in PSA reaction. We have previously shown that daily rhythms observed in passive cutaneous anaphylactic (PCA) reaction were absent in adrenalectomized mice [6], suggesting that humoral factors from the adrenal gland are necessary to maintain the daily rhythms. The central SCN pacemaker primarily governs the daily rhythmic secretions of humoral factors such as corticosterone from the adrenal gland [9]. Therefore, the SCN may control the time of day-dependent variation in PSA (and also PCA) reaction via regulation of the rhythmic secretion of humoral factors (possibly corticosterone) from the adrenal gland. The current findings that the time of day-dependent variation in PSA reaction in sham-operated mice showed an inverse association with the serum corticosterone levels and the extent of PSA reaction was severe in mice with disrupted SCN in association with low serum corticosterone levels may support this hypothesis.

In summary, the current findings provide additional support for the notion that the circadian clock system times IgE-mediated systemic anaphylactic reaction [6]. The findings may provide a novel insight into the pathophysiology of anaphylaxis and also implicate that environmental and intrinsic factors disrupting the normal activity of the circadian clock system, such as sleep disturbance and mental stress [5, 6, 10], might predispose some types of allergic patients (e.g., patients with drug and food allergy) to severe anaphylaxis.

## 4. Conclusions

The circadian clock may be an important regulator of anaphylaxis, which will provide a novel insight into the pathophysiology of the disease.
